# MHY1485 potentiates immunogenic cell death induction and anti-cancer immunity following irradiation

**DOI:** 10.1093/jrr/rrad107

**Published:** 2024-02-08

**Authors:** Lue Sun, Kumi Morikawa, Yu Sogo, Yuki Sugiura

**Affiliations:** Health and Medical Research Institute, Department of Life Science and Biotechnology, National Institute of Advanced Industrial Science and Technology (AIST), Central 6, 1-1-1 Higashi, Tsukuba, Ibaraki 305-8566, Japan; Health and Medical Research Institute, Department of Life Science and Biotechnology, National Institute of Advanced Industrial Science and Technology (AIST), Central 6, 1-1-1 Higashi, Tsukuba, Ibaraki 305-8566, Japan; Health and Medical Research Institute, Department of Life Science and Biotechnology, National Institute of Advanced Industrial Science and Technology (AIST), Central 6, 1-1-1 Higashi, Tsukuba, Ibaraki 305-8566, Japan; Health and Medical Research Institute, Department of Life Science and Biotechnology, National Institute of Advanced Industrial Science and Technology (AIST), 2217-14, Hayashi-cho, Takamatsu, Kagawa 761-0895, Japan

**Keywords:** MHY1485, radiation, anti-cancer immunity, DAMPs, immunogenic cell death

## Abstract

Recent *in vitro* experiments showed that combined treatment with MHY1485, a low-molecular-weight compound, and X-ray irradiation significantly increased apoptosis and senescence in tumor cells, which was associated with oxidative stress, endoplasmic reticulum (ER) stress and p21 stabilization, compared to radiation treatment alone. However, evidence for MHY1485 treatment-mediated suppression of tumor growth in animals is still lacking. Furthermore, it has been shown that ER stress enhances immunogenic cell death (ICD) in tumor cells, as it can exert a favorable influence on the anti-cancer immune system. In the present study, we examined whether co-treatment of MHY1485 and X-ray irradiation induces ICD and *in vivo* tumor growth suppression using the CT26 and Lewis lung carcinoma murine tumor cell lines. We found that MHY1485 + X-ray treatment promotes ICD more effectively than X-ray treatment alone. MHY1485 suppresses tumor growth *in vivo* under co-treatment with X-rays and increases INF-γ, tumor necrosis factor, interleukin-2 and interleukin-12 levels in the spleen as well as the presence of CD8+ cells in the tumor. The results suggest that MHY1485 treatment leads to the conversion of irradiated tumors into effective vaccines. Thus, MHY1485 is a promising lead compound for use in combination with radiotherapy.

## INTRODUCTION

The anti-cancer immune system is thought to play a very important role in improving post-radiotherapy prognosis. Apetoh *et al*. reported that radiotherapy reduced tumor growth and prolonged the survival of tumor-bearing immunocompetent wild-type mice, yet was less effective in immunodeficient mice [1]. Takeshima *et al*. reported that the inhibition of CD8+ T cells by administration of an anti-CD8 mAb into tumor-bearing mice negated radiation-induced tumor growth inhibition [2]. It is also known that adequate radiotherapy can enhance anti-tumor immune responses through immunogenic cell death (ICD) via the release of damage-associated molecular patterns and exposure of MHC Class I and calreticulin [2–4]. Moreover, radiation treatment is also able to downregulate anti-cancer immunity associated with increased tumor infiltration of immunosuppressive cells, such as myeloid-derived suppressive cells [5], regulatory T cells [6] and cancer-associated fibroblasts [7], and upregulate immunosuppressive molecules, such as programmed cell death ligand 1 (PD-L1) [8] and transforming growth factor-β (TGF-β) [9]. These findings suggest that radiotherapy exhibits a functional duality, delicately regulating the balance between immune inhibition and promotion. Similarly, several anti-cancer drugs, such as doxorubicin, can induce ICD [10], while other anti-cancer drugs (e.g. everolimus and others) have strong immunosuppressive effects [11]. Everolimus showed a marked radiosensitizing ability in an immunodeficient mouse tumor model [12]. However, this combination therapy failed in clinical trials [13], which highlights the importance of evaluating cytotoxicity as well as the effects on immunity when developing radiosensitizers.

MHY1485 is a low-molecular-weight compound that has an inhibitory effect on autophagy and activates the mechanistic target of rapamycin [14]. Sun *et al*. have reported that MHY1485 exhibits radiosensitizing activity and that the combination of MHY1485 and radiation treatment induced higher endoplasmic reticulum (ER) stress than radiation treatment alone *in vitro* [15]. ER stress is defined as a perturbation impacting ER homeostasis and is primarily characterized by the accumulation of aberrant proteins [16]. The unfolded protein response (UPR) is a mechanism for detecting and removing aberrant proteins to achieve ER homeostasis, which is partly mediated via the activation of autophagy [17]. However, an excessive accumulation of aberrant proteins induced by ionizing radiation or certain anti-cancer drugs promotes apoptotic cell death via the UPR [18, 19]. It has been reported that ER stress loading on cancer cells enhances anti-tumor immunity by generating ‘find-me’ and ‘eat-me’ signals such as adenosine triphosphate (ATP) release and increased membrane expression of calreticulin via the activation of protein kinase ribonucleic acid-like endoplasmic reticulum kinase endoplasmic reticulum kinase [20]. Therefore, in this study, we investigated the potential of MHY1485 to enhance the radiation-induced ICD. Further, we used a radiotherapy allogeneic model to determine whether MHY1485 inhibits tumor growth and affects anti-tumor immunity.

## MATERIALS AND METHODS

### Reagents and irradiation

MHY1485 was obtained from Selleck Chemicals (Tokyo, Japan). A CP-160 X-ray generator (Faxitron, AZ, USA) was used for X-ray irradiation. Irradiation was performed using a tube voltage of 160 kV, a tube current of 6.2 mA, filters of 0.2 mm Cu and 0.5 mm Al. The dose rate was ~0.92 Gy/min.

### Cell line and culture conditions

The murine colon carcinoma cell line CT26 (American Type Culture Collection, VA, USA) and the Lewis lung carcinoma (LLC) cell line (Riken Bioresource Center, Ibaraki, Japan) were cultured as described previously [15]. CT26 cells express the gp70423–431 (AH1) antigen [21] and are known as a ‘hot tumor’ that readily responds to immunotherapy [22]. To the best of our knowledge, LLC cells do not have a distinctive antigen and are known as a ‘cold tumor’ that responds poorly to immunotherapy [23]. CT26 cells showed higher MHC Class I expression and lower PD-L1 levels than LLC cells (Supplementary Fig. 1).

The number of cells was counted with a Countess II FL (Thermo Fisher Scientific, Tokyo, Japan). According to a previous report [15], the cells were plated in culture flasks or dishes at Day 0; 10 μM of MHY1485 or vehicle control (dimethyl sulfoxide (DMSO)) were added on Day 1 (24 h after cell plating); 6 Gy X-ray irradiation was delivered on Day 2 (24 h after drug treatment) and the culture medium and cells were collected for experiments on Day 3 (24 h after irradiation).

### ATP and HMGB1 production analysis

ATP and HMGB1 levels in the medium were measured using a CellTiter-Glo® Luminescent Cell Viability Assay (Promega, WI, USA) and an HMGB1 Measuring Kit FUSO (Fuso Pharmaceutical Industries, Osaka, Japan), respectively, according to the recommended protocol. Luminescent intensity and absorbance (OD 450 nm) were measured using an MTP-900Lab plate reader (Hitachi High-Tech Science Corporation, Tokyo, Japan).

### Cell surface calreticulin, MHC Class I (H-2Kd in murine models) and PD-L1 expression analysis

The cells were trypsinized and stained for 30 min with PE anti-mouse calreticulin (Cat. ab83220; Abcam, Cambridge, UK), H-2Kd (Cat. 116 607; Biolegend, CA, USA) and PD-L1 antibodies (Cat. 124 307, Biolegend). Dead cells were detected using propidium iodide (FUJIFILM Wako Pure Chemical Corporation, Osaka, Japan) and were excluded from the analysis. The median fluorescence intensity (MFI) was analyzed by flow cytometry using a BD Accuri C6 Plus (BD Biosciences, CA, USA) [24].

### Phosphorylated H2AX analysis

It has been reported that radiation induces MHC Class I and PD-L1 expression via the deoxyribonucleic acid (DNA) damage response pathway [25, 26]. Thus, we analyzed the phosphorylated H2AX (γH2AX levels), a marker of DNA double-strand breaks (DSB), according to previous methods [15]. Briefly, cells were trypsinized and fixed in 4% paraformaldehyde (FUJIFILM Wako) for 10 min, then permeabilized in Permeabilization Wash Buffer (BioLegend, San Diego, CA, USA). The cells were then sequentially incubated with anti-phospho-histone H2AX (Ser139) antibody (Merck KGaA, Darmstadt, Germany) and an Alexa Fluor 488-conjugated secondary antibody (Abcam). The MFI was analyzed using a BD Accuri C6 Plus [24].

### Mice and treatment

The vaccination-rechallenge assay is considered as the gold standard for evaluating ICD [27]. In this assay, cancer cells are inactivated *in vitro* and are administered as a vaccine to mice. The untreated (healthy) cancer cells are subsequently transplanted into the mice and the rate of tumor growth is evaluated. If the inactivated cancer cells function as a vaccine, the tumor growth rate is reduced or no tumor is produced at all. For the vaccination-rechallenge assay, CT26 and LLC cells were treated with 10 μM of MHY1485 or DMSO 24 h before being subjected to 20 Gy X-ray irradiation *in vitro*. The 20 Gy dose used in this study was based on a previous study [28]. The cells were trypsinized and suspended in Ca^++^- and Mg^++^-free phosphate-buffered saline (PBS; FUJIFILM Wako) 24 h after irradiation. Irradiated CT26 and LLC cells (1 × 10^5^) were vaccinated (subcutaneously injected into the right flank area) to male BALB/c and female C57BL/6 J mice (CLEA Japan, Tokyo, Japan), respectively. Ten days after injection, healthy CT26 (6 × 10^5^ cells/mouse) and LLC (3 × 10^5^ cells/mouse) cells were implanted (subcutaneously injected into the left flank area) in the mice, respectively.

For the therapeutic model, CT26 (6 × 10^5^ cells/mouse) and LLC (5 × 10^5^ cells/mouse) cells were subcutaneously implanted in the left hind leg of male BALB/c and female C57BL/6 J mice, respectively. When the tumor size reached ~100 mm^3^, the mice were treated with MHY1485 and irradiation. The tumor size of 100 mm^3^ was selected in this study based on a previous study [29]. MHY1485 (15 or 30 mg/kg) or vehicle control (DMSO) were mixed with corn oil and injected into the tumors twice (24 h between injections). Combination therapy was provided 3 h after the last MHY1485 injection. Anesthetized mice were locally irradiated with an X-ray dose of 8 Gy. The 8 Gy dose was selected based on a previous study [30]. Mice were anesthetized during radiation exposure using a mixture of anesthetic agents (0.75 mg/kg of medetomidine (Nippon Zenyaku Kogyo Co., Fukushima, Japan), 4.0 mg/kg of midazolam (Maruishi Pharmaceutical Co., Osaka, Japan) and 5.0 mg/kg of butorphanol (Meiji Seika Pharma Co., Tokyo, Japan). In addition, 0.75 mg/kg of atipamezole (Nippon Zenyaku Kogyo Co.) was administered after radiation exposure to reverse the effects of medetomidine [31].

The tumor length and width were measured using calipers. Tumor size was calculated using the equation *V* = (*a* × *b*^2^)/2, where *V* is the tumor volume, *a* is the length and *b* is the width of the tumor. According to the animal welfare requirements, the time required to observe a tumor volume of 1500 mm^3^ was designated as the survival period. Animals were euthanized when their tumor size exceeded 1500 mm^3^.

### Cytokine levels in spleen

The mice were sacrificed by cervical dislocation and spleens were collected when the mice met the endpoint conditions (tumor size: 1500 mm^3^ or >60 days after tumor cell transplantation) in the therapeutic model. Spleen tissues (100 mg) were homogenized and suspended in 1 ml of RPMI1640 medium. Interferon-gamma, tumor necrosis factor (TNF), interleukin-2 (IL-2) and interleukin-12 (IL-12p70) levels were measured using a Cytometric Bead Array (BD Biosciences) according to the manufacturer’s protocols.

### Immunofluorescence staining

The tumors were collected 7 days after X-ray irradiation, mounted in Optimal Cutting Temperature Compound (Sakura Finetek Japan, Tokyo, Japan) and frozen at −80°C. The frozen tissue was sliced into 8-μm thick sections using a cryostat (Tissue-Tek Polar DM; Sakura Finetek Japan) and was placed on precoated glass slides (Matsunami Glass, Osaka, Japan). The tissue sections were stored at −80°C until staining.

After drying at room temperature, the tumor sections were fixed with 4% paraformaldehyde solution (FUJIFILM Wako) for 10 min at room temperature. The tumor sections were washed three times with PBS for 5 min each at room temperature. The tumor sections were blocked with 10% goat serum (FUJIFILM Wako) in PBS with 0.5% Tween-20 (FUJIFILM Wako) overnight at 4°C. The tumor sections were then incubated overnight at 4°C with a 1:500 dilution of anti-mouse CD8α antibody (Cat. 98 941; Cell Signaling Technology, MA, USA) in PBS with 1% goat serum buffer. The cells were then incubated for 1 h at room temperature with a 1:500 dilution of Alexa Fluor 488-conjugated goat anti-rabbit IgG secondary antibody (Abcam). The cells were counterstained with 4′,6-diamidino-2-phenylindole (DAPI), mounted using a TrueVIEW™ Autofluorescence Quenching Kit with DAPI (Vector Laboratories, CA, USA) and viewed using a fluorescence microscope (BZ-X700; Keyence, Osaka, Japan). A minimum of five images were obtained from each tumor and the number of CD8+ cells per mm^2^ was determined.

### Statistical analysis

The mean and standard deviation (SD) were calculated for each quantitative parameter. A two-tailed two-way analysis of variance (ANOVA) with Sidak’s multiple comparisons *post hoc* test was used for comparing the statistical differences between multiple groups. A two-tailed Student’s *t*-test was used for comparing the statistical differences between two groups. A log-rank test was used to compare the survival distributions. A *P*-value of <0.05 was considered to be statistically significant.

## RESULTS

### MHY1485 enhances radiation-induced ICD

**Fig. 1:**
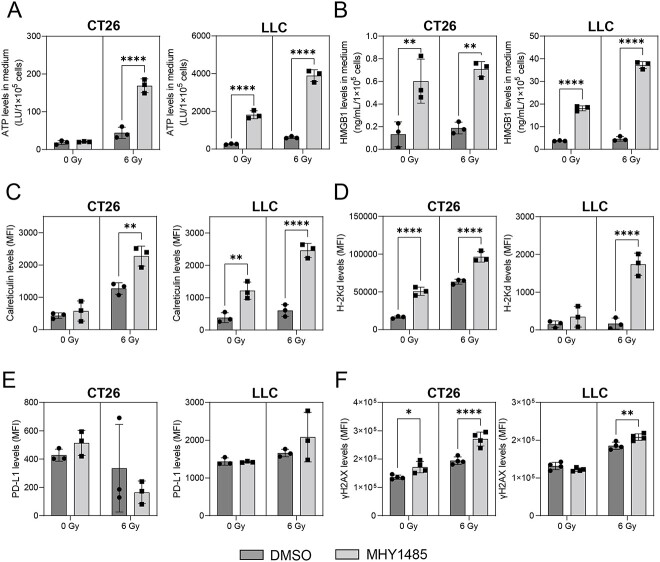
Expression of ICD markers CT26 and LLC cells. (**A**) ATP and (**B**) HMGB1 levels were determined in medium. Cell surface (**C**) calreticulin, (**D**) H-2Kd, (**E**) PD-L1 and (**F**) γH2AX expression levels in CT26 and LLC cells. All quantitative data are presented as mean ± SD (*n* = 3–4). ^*^P < 0.05, ^*^^*^P < 0.01, ^*^^*^^*^^*^P < 0.0001, two-way ANOVA with Sidak’s multiple comparisons *post hoc* test vs respective DMSO control.

First, we investigated whether MHY1485 treatment increases the expression of ICD-related molecules. In CT26 cells, MHY1485 treatment significantly increased HMGB1 release and cell surface H-2Kd expression in the absence of irradiation and significantly increased ATP and HMGB1 release and cell surface calreticulin and H-2Kd expression under irradiation (Fig. 1A–D). In LLC cells, MHY1485 treatment significantly increased ATP and HMGB1 release and cell surface calreticulin expression in the absence of irradiation and significantly increased ATP and HMGB1 release and cell surface calreticulin and H-2Kd expression under irradiation (Fig. 1A–D). These results suggest that MHY1485 enhances the expression levels of ICD markers, especially under irradiation. In addition, we analyzed the PD-L1 and DSB (γH2AX) levels, which are factors that affect the anti-tumor immunity after radiotherapy [25, 26]. We found that MHY1485 treatment did not alter the PD-L1 expression levels under both non-irradiated and irradiated conditions in both CT26 and LLC cells (Fig. 1E). In CT26 cells, MHY1485 treatment significantly increased DSB (γH2AX) levels in both the absence and presence of irradiation (Fig. 1F). In LLC cells, MHY1485 treatment significantly increased the DSB (γH2AX) levels under irradiation only (Fig. 1F).

**Fig. 2:**
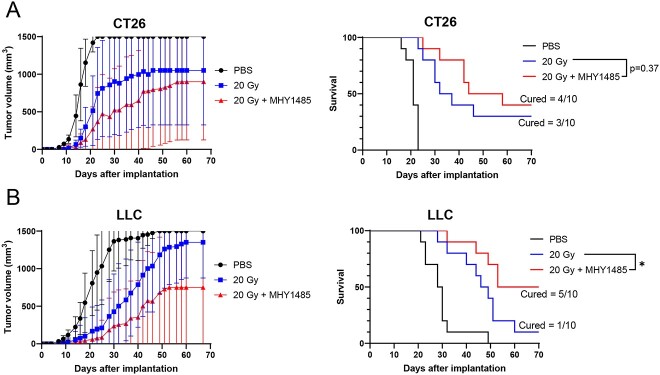
Tumor growth and survival curves of the vaccination-rechallenge model. (**A**) CT26 and (**B**) LLC tumor models (*n* = 10 for each group). The mice received subcutaneous injections of PBS (no cells), *in vivo* 20 Gy irradiated tumor cells and *in vivo* MHY1485 treated and 20 Gy irradiated tumor cells as tumor vaccines. Healthy tumor cells were subsequently injected subcutaneously and tumor sizes were measured. The time required to develop tumors of 1500 mm^3^ was determined as the survival period. We substituted the value of 1500 mm^3^ when tumor size exceeded 1500 mm^3^. The symbols indicate average tumor volume, and the error bars indicate SD for tumor growth curves. Significant differences between the irradiated tumor cell vaccine groups and co-treated MHY1485 and radiation tumor cell vaccine groups were analyzed using the two-tailed Student’s *t*-test or log-rank test, ^*^P < 0.05.

We then performed a vaccination-rechallenge assay for analyzing ICD. Both the CT26 and LLC models showed large differences in tumor growth rates even within the same group (in particular, mice that failed to develop tumors contributed to the increase in SD) (Supplementary Fig. 2 and Fig. 2). We were unable to identify significant differences in the tumor volume analysis (Fig. 2). Hence, instead of the average tumor volume, the time required for the tumor volume to reach 1500 mm^3^ was used as the analysis index. We found that the MHY1485 and radiation co-treated tumor cell vaccine group did not prevent rechallenged tumor growth as compared with the irradiation-only tumor cell vaccine group when using CT26 cells (Fig. 2A). However, the MHY1485 and radiation co-treated tumor cell vaccine group significantly reduced rechallenged tumor growth as compared with the irradiation-only tumor cell vaccine group with LLC cells (Fig. 2B). These results suggest that MHY1485 potentially enhances radiation-induced ICD.

### Treatment with MHY1485 potentiates radiotherapy and increases anti-cancer immunity in tumor allogeneic models

**Fig. 3:**
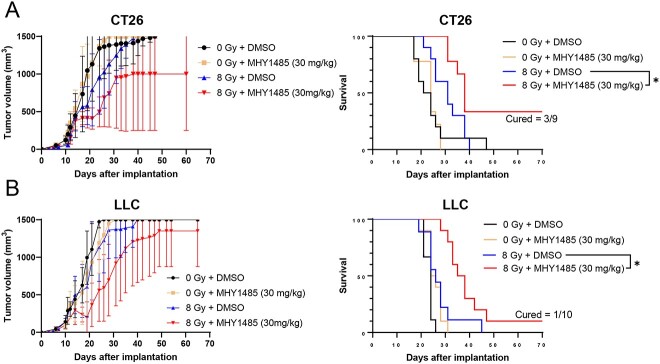
Tumor growth and survival curves of the therapeutic model. (**A**) CT26 and (**B**) LLC tumor models (*n* = 9–10). In the CT26 model, 30 mg/kg of MHY1485 was administered on Days 12 and 13, and 8 Gy irradiation was delivered on Day 13. In the LLC model, 30 mg/kg of MHY1485 was administered on Days 10 and 11, and 8 Gy irradiation was delivered on Day 11. The time required to develop tumors of 1500 mm^3^ was determined as the survival period. We substituted the value of 1500 mm^3^ when the tumor size exceeded 1500 mm^3^. The symbols indicate average tumor volume, and the error bars indicate SD for tumor growth curves. Significant differences between radiation + DMSO groups and radiation + MHY1485 groups were analyzed using the two-tailed Student’s *t*-test or log-rank test, ^*^P < 0.05.

**Fig. 4:**
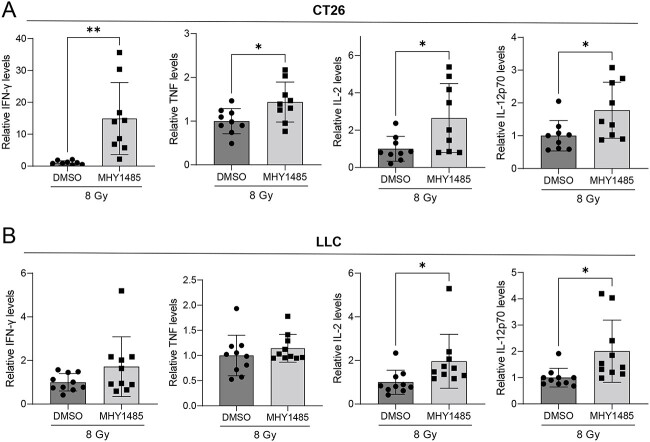
Cytokine levels in spleen. Spleens from therapeutic model mice were collected from the radiation + DMSO and radiation + MHY1485 groups. (**A**) CT26 and (**B**) LLC (*n* = 9–10). ^*^P < 0.05, ^*^^*^P < 0.01, two-tailed Student’s *t*-test.

**Fig. 5:**
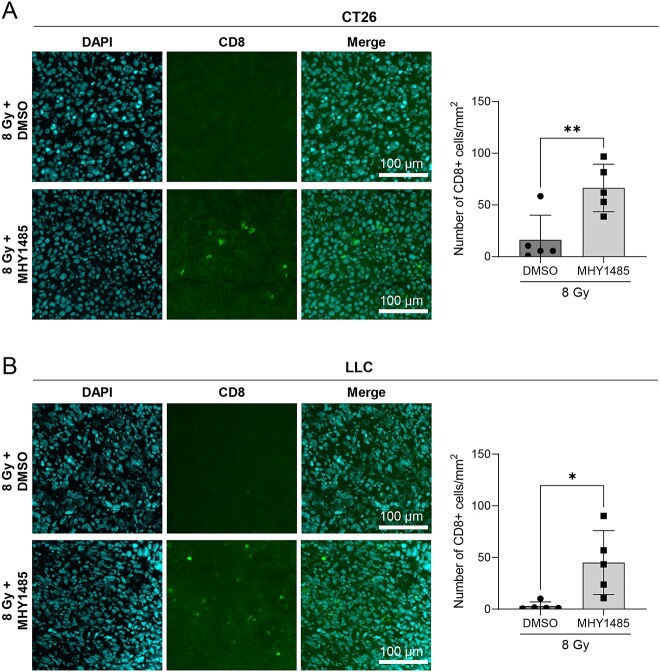
CD8+ cells in tumors. Tumors from therapeutic model mice were collected from the radiation + DMSO and radiation + MHY1485 groups 7 days after irradiation. (**A**) CT26 and (**B**) LLC (*n* = 5). Five images or more were obtained from each tumor and the numbers of CD8+ cells per mm^2^ were determined. ^*^P < 0.05, ^*^^*^P < 0.01, two-tailed Student’s *t*-test.

We further investigated whether MHY1485 treatment inhibits tumor growth in a therapeutic model. As with the vaccination-rechallenge experiment, the time required for tumor volume to reach 1500 mm^3^ was used as the analysis index. In both the CT26 and LLC models, the groups administered with 30 mg/kg MHY1485 alone showed no tumor growth suppression in comparison with the DMSO group; by contrast, the group administered with 30 mg/kg MHY1485 with radiation showed significant tumor growth suppression as compared to the radiation + DMSO group (Fig. 3 and Supplementary Fig. 3). However, tumor growth suppression was not observed in animals receiving 15 mg/kg MHY1485 (Supplementary Fig. 4). Further, the radiation +30 mg/kg MHY1485-treated group showed higher INF-γ, TNF, IL-2 and IL-12p70 levels in the spleen in comparison to the radiation + DMSO-treated group in the CT26 model (Fig. 4A). The radiation +30 mg/kg MHY1485-treated group showed higher IL-2 and IL-12p70 levels in the spleen in comparison to the radiation + DMSO-treated group in the LLC model (Fig. 4B). In both the CT26 and LLC models, a high percentage of CD8+ cells were observed in the radiation +30 mg/kg MHY1485-treated tumors as compared with the radiation + DMSO-treated tumors (Fig. 5). These results indicate that MHY1485 enhances radiation-induced tumor growth suppression and anti-cancer immune responses *in vivo*.

## DISCUSSION

Sun *et al*. found that MHY1485 produced a radiosensitizing effect *in vitro*, which was partly associated with ER and oxidative stress overloading [15]. In this study, we analyzed MHY1485’s radiosensitizing effect using a tumor-bearing mouse model and investigated whether MHY1485 treatment affects the anti-tumor immune system. We found that combination treatment of MHY1485 with radiation increased the expression of ICD markers (such as ATP and HMGB1 secretion, calreticulin and MHC Class I expression) in comparison to radiation-alone treatment (Fig. 1A–D). Extracellular ATP functions as a prominent ‘find-me’ signal for dendritic cell (DC) precursors and macrophages [32]. HMGB1 accelerates the processing of phagocytic cargo in DCs and facilitates antigen presentation by DCs to T cells [33]. Surface-exposed calreticulin binds to CD91 expressed by antigen-presenting cells and promotes the engulfment of dead cells and debris [32]. CD8 T cells identify cancers by recognizing peptide–MHC Class I complexes that are generated through the MHC Class I antigen presentation pathway [34]. Together with previous studies, this study shows that MHY1485 promotes ICD marker expression and release, which may be associated with ER and oxidative stress overloading [15,35–38]. The activation of anti-tumor immunity induced from whole-cell vaccines is influenced by various immune activators and suppressors. For example, it has been reported that enhancing the GM-CSF and IL-2 release from whole-cell vaccines can improve their efficacy [39, 40]. It has also been reported that optimally irradiated apoptotic whole-cell vaccines have higher efficacy than freeze-thawed necrotic whole-cell vaccines [41]. This is thought to be because necrotic cells stimulate the release of immunosuppressive factors (IL-10 and TGF-β) from macrophages. In addition, the activation of DCs and T cells is essential for the activation of anti-tumor immunity [41]. These observations suggest that an increased expression of ICD markers may not directly lead to the activation of anti-tumor immunity. The present study showed that MHY1485 + radiation-treated whole-cell vaccines significantly delayed tumor growth as compared to whole-cell vaccines treated with radiation alone in the LLC model (Fig. 2B). By contrast, no significant difference was observed in the CT26 model (Fig. 2A). These results indicate that the degree of enhancement of radiation-induced ICD by MHY1485 varies depending on the type or nature of cancer and that further elucidation of the mechanism is needed.

In a therapeutic model, we found that MHY1485 treatment on its own did not affect tumor growth, whereas MHY1485 + radiation treatment significantly delays tumor growth in comparison to radiation treatment alone in both the CT26 and LLC models (Fig. 3). In addition, the MHY1485 + radiation group showed a higher CD8+ cell ratio in tumors and anti-cancer immunity-related cytokines than the radiation-alone group, suggesting that anti-cancer immunity was activated in the MHY1485 + radiation group (Figs 4 and 5). Interestingly, 40% of mice completely rejected the tumors in the MHY1485 + radiation group in the CT26 model, but only 10% of mice completed rejected the tumors in the MHY1485 + radiation group in the LLC model (Fig. 3). An analysis of cytokine levels indicated that all four cytokines (INF-γ, TNF, IL-2 and IL-12p70) were upregulated in the MHY1485 + radiation group in the CT26 model, but only two cytokines (IL-2 and IL-12p70) were upregulated in the MHY1485 + radiation group in the LLC model (Fig. 4). Our results suggest that MHY1485 was capable of activating the anti-cancer immune system when combined with radiotherapy.

This study found that MHY1485 is active as a radiosensitizing agent. Further, our results suggest that MHY1485 enhances anti-tumor immunity associated with the induction of ICD. However, to achieve these effects *in vivo*, high concentrations, such as a 30 mg/kg intratumor (i.t.) injection, are required. Intraperitoneal and intravenous administrations are the common administration routes for antitumor drugs in clinical practice; however, i.t. administration allows for higher intra-tumoral drug concentrations and may reduce systemic side effects [42]. Thus, it may be difficult to achieve the same therapeutic effect using intraperitoneal and intravenous administration. Moreover, it is possible that MHY1485 can be used as a lead compound for synthesizing more powerful radiosensitizers since it has been reported that ICD inducers are rare compounds, with only ~5% of FDA-approved anti-cancer drugs capable of stimulating ICD [43]. However, the detailed molecular mechanism of ICD induction by MHY1485 remains unclear, and our findings are somewhat inconsistent with previous reports. We and Sun *et al*. showed that MHY1485 + radiation treatment increased the oxidative stress, DSB levels, MHC Class I expression and ER stress loading but that it did not affect the PD-L1 levels as compared with radiation treatment alone (Fig. 1D–F) [15]. It has been reported that radiation-induced DNA damage promotes ER stress and HLA Class I presentation, while Ulianich *et al*. showed that ER stress loading decreases MHC Class I expression [26, 44, 45]. Moreover, it has been reported that the DNA DSB repair pathway and oxidative stress regulate PD-L1 expression [25, 46]. These inconsistencies should be clarified in future studies.

## CONCLUSION

MHY1485 treatment led to the conversion of irradiated tumors into effective vaccines. Moreover, MHY1485 may be a promising lead compound for use in combination with radiotherapy.

## CONFLICT OF INTEREST

The authors declare no conflicts of interest associated with this manuscript.

## ETHICAL CONSIDERATIONS

All animal experiments were performed in accordance with the National Institute of Advanced Industrial Science and Technology (AIST). All animal husbandry procedures and experiments were approved by the Animal Experiment Committee of AIST (Permit Number: 2021-0283 and 2021-0381).

## Supplementary Material

Supplementary_fig_rrad107

## References

[1] Apetoh L , Ghiringhelli F, Tesniere A, et al. Toll-like receptor 4-dependent contribution of the immune system to anticancer chemotherapy and radiotherapy. Nat Med 2007;13:1050–9. 10.1038/nm1622. 17704786

[2] Takeshima T , Chamoto K, Wakita D, et al. Local radiation therapy inhibits tumor growth through the generation of tumor-specific CTL: its potentiation by combination with Th1 cell therapy. Cancer Res 2010;70:2697–706. 10.1158/0008-5472.CAN-09-2982. 20215523

[3] Onishi M , Okonogi N, Oike T, et al. High linear energy transfer carbon-ion irradiation increases the release of the immune mediator high mobility group box 1 from human cancer cells. J Radiat Res 2018;59:541–6. 10.1093/jrr/rry049. 29947767 PMC6151640

[4] Zeng H , Zhang W, Gong Y, Xie C. Radiotherapy activates autophagy to increase CD8(+) T cell infiltration by modulating major histocompatibility complex class-I expression in non-small cell lung cancer. J Int Med Res 2019;47:3818–30. 10.1177/0300060519855595. 31187666 PMC6726798

[5] Vatner RE , Formenti SC. Myeloid-derived cells in tumors: effects of radiation. Semin Radiat Oncol 2015;25:18–27. 10.1016/j.semradonc.2014.07.008. 25481262

[6] Muroyama Y , Nirschl TR, Kochel CM, et al. Stereotactic radiotherapy increases functionally suppressive regulatory T cells in the tumor microenvironment. Cancer Immunol Res 2017;5:992–1004. 10.1158/2326-6066.CIR-17-0040. 28970196 PMC5793220

[7] Wang Z , Tang Y, Tan Y, et al. Cancer-associated fibroblasts in radiotherapy: challenges and new opportunities. Cell Commun Signal 2019;17:47. 10.1186/s12964-019-0362-2. 31101063 PMC6525365

[8] Gong J , Le TQ, Massarelli E, et al. Radiation therapy and PD-1/PD-L1 blockade: the clinical development of an evolving anticancer combination. J Immunother Cancer 2018;6:46. 10.1186/s40425-018-0361-7. 29866197 PMC5987486

[9] Wang J , Xu Z, Wang Z, et al. TGF-beta signaling in cancer radiotherapy. Cytokine 2021;148:155709. 10.1016/j.cyto.2021.155709. 34597918

[10] Wang YJ , Fletcher R, Yu J, Zhang L. Immunogenic effects of chemotherapy-induced tumor cell death. Genes Dis 2018;5:194–203. 10.1016/j.gendis.2018.05.003. 30320184 PMC6176216

[11] Patel JK , Kobashigawa JA. Everolimus: an immunosuppressive agent in transplantation. Expert Opin Pharmacother 2006;7:1347–55. 10.1517/14656566.7.10.1347. 16805720

[12] Mauceri HJ , Sutton HG, Darga TE, et al. Everolimus exhibits efficacy as a radiosensitizer in a model of non-small cell lung cancer. Oncol Rep 2012;27:1625–9. 10.3892/or.2012.1666. 22294050

[13] Ma DJ , Galanis E, Anderson SK, et al. A phase II trial of everolimus, temozolomide, and radiotherapy in patients with newly diagnosed glioblastoma: NCCTG N057K. Neuro-Oncology 2015;17:1261–9. 10.1093/neuonc/nou328. 25526733 PMC4588750

[14] Choi YJ , Park YJ, Park JY, et al. Inhibitory effect of mTOR activator MHY1485 on autophagy: suppression of lysosomal fusion. PLoS One 2012;7:e43418. 10.1371/journal.pone.0043418. 22927967 PMC3425474

[15] Sun L , Morikawa K, Sogo Y, Sugiura Y. MHY1485 enhances X-irradiation-induced apoptosis and senescence in tumor cells. J Radiat Res 2021;62:782–92. 10.1093/jrr/rrab057. 34265852 PMC8438247

[16] Thangaraj A , Sil S, Tripathi A, et al. Targeting endoplasmic reticulum stress and autophagy as therapeutic approaches for neurological diseases. Int Rev Cell Mol Biol 2020;350:285–325. 10.1016/bs.ircmb.2019.11.001. 32138902

[17] Dara L , Ji C, Kaplowitz N. The contribution of endoplasmic reticulum stress to liver diseases. Hepatology 2011;53:1752–63. 10.1002/hep.24279. 21384408 PMC3082587

[18] Yoshino H , Kumai Y, Kashiwakura I. Effects of endoplasmic reticulum stress on apoptosis induction in radioresistant macrophages. Mol Med Rep 2017;15:2867–72. 10.3892/mmr.2017.6298. 28447729

[19] Bagchi AK , Malik A, Akolkar G, et al. Study of ER stress and apoptotic proteins in the heart and tumor exposed to doxorubicin. Biochim Biophys Acta Mol Cell Res 2021;1868:119039. 10.1016/j.bbamcr.2021.119039. 33857568

[20] Kepp O , Menger L, Vacchelli E, et al. Crosstalk between ER stress and immunogenic cell death. Cytokine Growth Factor Rev 2013;24:311–8. 10.1016/j.cytogfr.2013.05.001. 23787159

[21] Jordan KR , McMahan RH, Kemmler CB, et al. Peptide vaccines prevent tumor growth by activating T cells that respond to native tumor antigens. Proc Natl Acad Sci U S A 2010;107:4652–7. 10.1073/pnas.0914879107. 20133772 PMC2842066

[22] Sato Y , Fu Y, Liu H, et al. Tumor-immune profiling of CT-26 and colon 26 syngeneic mouse models reveals mechanism of anti-PD-1 response. BMC Cancer 2021;21:1222. 10.1186/s12885-021-08974-3. 34774008 PMC8590766

[23] Bates AM , Brown RJ, Pieper AA, et al. Combination of bempegaldesleukin and anti-CTLA-4 prevents metastatic dissemination after primary resection or radiotherapy in a preclinical model of non-small cell lung cancer. Front Oncol 2021;11:645352. 10.3389/fonc.2021.645352. 33937052 PMC8083981

[24] Permata TBM , Sato H, Gu W, et al. High linear energy transfer carbon-ion irradiation upregulates PD-L1 expression more significantly than X-rays in human osteosarcoma U2OS cells. J Radiat Res 2021;62:773–81. 10.1093/jrr/rrab050. 34196706 PMC8438258

[25] Sato H , Niimi A, Yasuhara T, et al. DNA double-strand break repair pathway regulates PD-L1 expression in cancer cells. Nat Commun 2017;8:1751. 10.1038/s41467-017-01883-9. 29170499 PMC5701012

[26] Uchihara Y , Permata TBM, Sato H, et al. DNA damage promotes HLA class I presentation by stimulating a pioneer round of translation-associated antigen production. Mol Cell 2022;82:2557–70.e7. 10.1016/j.molcel.2022.04.030. 35594857

[27] Jin MZ , Wang XP. Immunogenic cell death-based cancer vaccines. Front Immunol 2021;12:697964. 10.3389/fimmu.2021.697964. 34135914 PMC8200667

[28] Zenkoh J , Gerelchuluun A, Wang Y, et al. The abscopal effect induced by in situ-irradiated peripheral tumor cells in a murine GL261 brain tumor model. Transl Cancer Res 2017;6:136–48. 10.21037/tcr.2017.01.32.

[29] Shibata Y , Yasui H, Higashikawa K, et al. Erastin, a ferroptosis-inducing agent, sensitized cancer cells to X-ray irradiation via glutathione starvation in vitro and in vivo. PLoS One 2019;14:e0225931. 10.1371/journal.pone.0225931. 31800616 PMC6892486

[30] Wang Y , Zenkoh J, Gerelchuluun A, et al. Administration of dendritic cells and anti-PD-1 antibody converts X-ray irradiated tumors into effective in situ vaccines. Int J Radiat Oncol Biol Phys 2019;103:958–69. 10.1016/j.ijrobp.2018.11.019. 30458232

[31] Sun L , Inaba Y, Sogo Y, et al. Analysis of whole-blood antioxidant capacity after chronic and localized irradiation using the i-STrap method. J Radiat Res 2022;63:30–5. 10.1093/jrr/rrab099. 34718686 PMC8776686

[32] Fucikova J , Kepp O, Kasikova L, et al. Detection of immunogenic cell death and its relevance for cancer therapy. Cell Death Dis 2020;11:1013. 10.1038/s41419-020-03221-2. 33243969 PMC7691519

[33] Yamazaki T , Hannani D, Poirier-Colame V, et al. Defective immunogenic cell death of HMGB1-deficient tumors: compensatory therapy with TLR4 agonists. Cell Death Differ 2014;21:69–78. 10.1038/cdd.2013.72. 23811849 PMC3857617

[34] Dhatchinamoorthy K , Colbert JD, Rock KL. Cancer immune evasion through loss of MHC class I antigen presentation. Front Immunol 2021;12:636568. 10.3389/fimmu.2021.636568. 33767702 PMC7986854

[35] Garg AD , Krysko DV, Verfaillie T, et al. A novel pathway combining calreticulin exposure and ATP secretion in immunogenic cancer cell death. EMBO J 2012;31:1062–79. 10.1038/emboj.2011.497. 22252128 PMC3298003

[36] Panaretakis T , Kepp O, Brockmeier U, et al. Mechanisms of pre-apoptotic calreticulin exposure in immunogenic cell death. EMBO J 2009;28:578–90. 10.1038/emboj.2009.1. 19165151 PMC2657583

[37] Tang D , Kang R, Zeh HJ 3rd, et al. High-mobility group box 1, oxidative stress, and disease. Antioxid Redox Signal 2011;14:1315–35. 10.1089/ars.2010.3356. 20969478 PMC3048826

[38] Collett GP , Redman CW, Sargent IL, et al. Endoplasmic reticulum stress stimulates the release of extracellular vesicles carrying danger-associated molecular pattern (DAMP) molecules. Oncotarget 2018;9:6707–17. 10.18632/oncotarget.24158. 29467921 PMC5805507

[39] Huang X , Ye D, Thorpe PE. Enhancing the potency of a whole-cell breast cancer vaccine in mice with an antibody-IL-2 immunocytokine that targets exposed phosphatidylserine. Vaccine 2011;29:4785–93. 10.1016/j.vaccine.2011.04.082. 21557977

[40] Keenan BP , Jaffee EM. Whole cell vaccines—past progress and future strategies. Semin Oncol 2012;39:276–86. 10.1053/j.seminoncol.2012.02.007. 22595050 PMC3356993

[41] Scheffer SR , Nave H, Korangy F, et al. Apoptotic, but not necrotic, tumor cell vaccines induce a potent immune response in vivo. Int J Cancer 2003;103:205–11. 10.1002/ijc.10777. 12455034

[42] Melero I , Castanon E, Alvarez M, et al. Intratumoural administration and tumour tissue targeting of cancer immunotherapies. Nat Rev Clin Oncol 2021;18:558–76. 10.1038/s41571-021-00507-y. 34006998 PMC8130796

[43] Kepp O , Senovilla L, Kroemer G. Immunogenic cell death inducers as anticancer agents. Oncotarget 2014;5:5190–1. 10.18632/oncotarget.2266. 25114034 PMC4170601

[44] Suzuki K , Gerelchuluun A, Hong Z, et al. Celecoxib enhances radiosensitivity of hypoxic glioblastoma cells through endoplasmic reticulum stress. Neuro-Oncology 2013;15:1186–99. 10.1093/neuonc/not062. 23658321 PMC3748914

[45] Ulianich L , Terrazzano G, Annunziatella M, et al. ER stress impairs MHC class I surface expression and increases susceptibility of thyroid cells to NK-mediated cytotoxicity. Biochim Biophys Acta 2011;1812:431–8. 10.1016/j.bbadis.2010.12.013. 21199669

[46] Bailly C . Regulation of PD-L1 expression on cancer cells with ROS-modulating drugs. Life Sci 2020;246:117403. 10.1016/j.lfs.2020.117403. 32035131

