# Supplementary material for: MHY1485 potentiates immunogenic cell death induction and anti-cancer immunity following irradiation
Source: J Radiat Res. 2024 Feb 8;65(2):205–14. doi: 10.1093/jrr/rrad107 (PMC10959436; doi:10.1093/jrr/rrad107)
Supplement: Supplementary_fig_rrad107 [file supplementary_fig_rrad107.docx]

**Supplementary fig. 1 MHC class I and Pd-L1 levels in CT26 and LLC cells.** (A) PD-L1 and (B) MHC class I (H-2Kd). All quantitative data are presented as mean ± SD (n = 3).

**Supplementary fig. 2 Tumor growth curves in the vaccination-rechallenge model.** The data correspond to Fig. 2. PBS: pre-treated with phosphate-buffered saline. 20 Gy: pre-treated with *in vivo* 20 Gy irradiated tumor cell vaccine. 20 Gy + MHY1485: pre-treated with *in vivo* MHY1485 and 20 Gy irradiated tumor cell vaccine. Tumor size endpoint is 1500 mm^3^. n = 10 for each group.

**Supplementary fig. 3 Tumor growth curves in the therapeutic model.** The data correspond to Fig. 3. In the CT26 model, 30 mg/kg of MHY1485 was administered on days 12 and 13 and 8 Gy irradiation was delivered on day 13. In the LLC model, 30 mg/kg of MHY1485 was administered on days 10 and 11 and 8 Gy irradiation was delivered on day 11. Tumor size endpoint is 1500 mm^3^. n = 9-10 for each group.
